# What Do the Transcriptome and Proteome of Menstrual Blood-Derived Mesenchymal Stem Cells Tell Us about Endometriosis?

**DOI:** 10.3390/ijms231911515

**Published:** 2022-09-29

**Authors:** Letícia B. C. Penariol, Carolina H. Thomé, Patrícia A. Tozetti, Carlos R. K. Paier, Fabiana O. Buono, Kamila C. Peronni, Maristela D. Orellana, Dimas T. Covas, Maria E. A. Moraes, Wilson A. Silva, Júlio C. Rosa-e-Silva, Rui A. Ferriani, Vitor M. Faça, Omero B. Poli-Neto, Daniel G. Tiezzi, Juliana Meola

**Affiliations:** 1 Department of Gynecology and Obstetrics, Medical School of Ribeirão Preto, University of São Paulo, São Paulo 14049-900, Brazil; 2 Regional Blood Center, Medical School of Hemocenter Foundation of Ribeirão Preto, University of São Paulo, São Paulo 14051-140, Brazil; 3 Drug Research and Development Center, Federal University of Ceara, Ceará 60430-275, Brazil; 4 Department of Genetics, Medical School of Ribeirão Preto, University of São Paulo, São Paulo 14049-900, Brazil; 5 Laboratory for Translational Data Science, Department of Gynecology and Obstetrics, Medical School of Ribeirão Preto, University of São Paulo, São Paulo 14049-900, Brazil; 6 National Institute of Hormones and Women’s Health (Hormona), CNPq, Porto Alegre 90035-003, Brazil; 7 Department Biochemistry and Immunology, Medical School of Ribeirão Preto, University of São Paulo, São Paulo 14049-900, Brazil

**Keywords:** endometriosis, multi-omics, expression profile, menstrual blood, MenSCs

## Abstract

Given the importance of menstrual blood in the pathogenesis of endometriosis and the multifunctional roles of menstrual mesenchymal stem cells (MenSCs) in regenerative medicine, this issue has gained prominence in the scientific community. Moreover, recent reviews highlight how robust the integrated assessment of omics data are for endometriosis. To our knowledge, no study has applied the multi-omics approaches to endometriosis MenSCs. This is a case-control study at a university-affiliated hospital. MenSCs transcriptome and proteome data were obtained by RNA-seq and UHPLC-MS/MS detection. Among the differentially expressed proteins and genes, we emphasize *ATF3, ID1, ID3, FOSB, SNAI1, NR4A1, EGR1, LAMC3,* and *ZFP36* genes and MT2A, TYMP, COL1A1, COL6A2, and NID2 proteins that were already reported in the endometriosis. Our functional enrichment analysis reveals integrated modulating signaling pathways such as epithelial–mesenchymal transition (↑) and PI3K signaling via AKT to mTORC1 (↓ in proteome), mTORC1 signaling, TGF beta signaling, TNFA signaling via NFkB, IL6 STAT3 signaling, and response to hypoxia via HIF1A targets (↑ in transcriptome). Our findings highlight primary changes in the endometriosis MenSCs, suggesting that the chronic inflammatory endometrial microenvironment can modulate these cells, providing opportunities for endometriosis etiopathogenesis. Moreover, they identify challenges for future research leveraging knowledge for regenerative and precision medicine in endometriosis.

## 1. Introduction

Menstrual blood is a non-invasive source for obtaining mesenchymal/stromal stem cells (MenSCs), which have a robust capacity for self-renewing, a high proliferation rate, pluripotency, and migratory and immunomodulatory functions in inflammatory, tumor, and tissue-injury conditions. Consequently, they are considered a promising tool for regenerative medicine [1,2,3]. Due to the varied potential of clinical applications combined with no ethical dilemma, MenSCs have gained prominence in the scientific community since their discovery in 2007 [4] in different gynecological diseases [5] and, therefore, in the context of endometriosis [6,7,8,9]. Particular emphasis has been given to the different functionalities of these progenitor cells regarding the etiopathogenic mechanism [8] and reproductive aspects of endometriosis [10].

Endometriosis is an enigmatic benign gynecological disease, estrogen-dependent, progesterone resistant, and chronically inflammatory, affecting 5–10% of women of reproductive age worldwide [11]. Its case history is heterogeneous, with lesions identified in 7% of asymptomatic women undergoing tubal ligation, 50% of adolescents with uncontrolled dysmenorrhea, 5–24% of women with persistent acyclic pain, and 10% to 40% of infertile women [12,13]. The disease is characterized histologically by endometrial tissue implants (glands and/or stroma) outside the uterine cavity (ectopic tissue), frequently located in the pelvis [14], and less commonly in the intestine, bladder, abdominal wall, thoracic cavity, and other organs [15,16]. Due to its impact on physical and psychological health as well as the socioeconomic impact on the costs of its diagnosis, treatment, and monitoring, endometriosis is considered a public health problem [17,18].

This characteristic heterogeneity of the disease is not only related to the clinical aspects but also its origin. Its etiopathogenesis is complex and not completely understood [19]. The source of the ectopic endometrium has been the subject of much investigation. Thus, several theories and hypotheses are suggested as concomitant [20]. In this sense, the idea that progenitor cells in the endometrium (eMSCs) and, consequently, present in the menstrual flow (MenSCs) are initiators and maintainers of ectopic lesions [21] makes up a very plausible hypothesis combined with the theory of Sampson’s retrograde menstruation [22].

Simultaneously with discovering these progenitor cells, several approaches such as genomics, epigenomics, transcriptomics, and proteomics have been applied to understand endometriosis [23,24,25,26]. Some differential gene or protein expression studies aimed at better understanding the gene expression behavior in stromal fibroblastic cells (SFs) and mesenchymal progenitors (eMSCs) were carried out using different experimental proposals in the endometrium, endometriotic lesions [27,28,29,30], and, more rarely, in menstrual flow cells [7,31]. Since the gene expression is modulated at the transcriptional, post-transcriptional, translational, and post-translational modifications, these intricate mechanisms often lead to inconclusive studies and arduous interpretations when a single “omic” is evaluated.

Recent reviews accentuate the importance of the “omics” era for endometriosis and highlight the powerful nature of the integrated assessment of these data [19,32]. Here, we describe for the first time the integrated pathways obtained from transcriptomic and proteomic data in endometriosis MenSCs that modulate biological processes involved in angiogenesis, proliferation, cell migration, and inflammatory response. We believe that dysregulated pathways may reflect primary alterations in these cells, favoring endometriosis.

## 2. Results

### 2.1. Study Flowchart, Clinical Variables, and the MenSCs In Vitro Model

**Figure 1:**
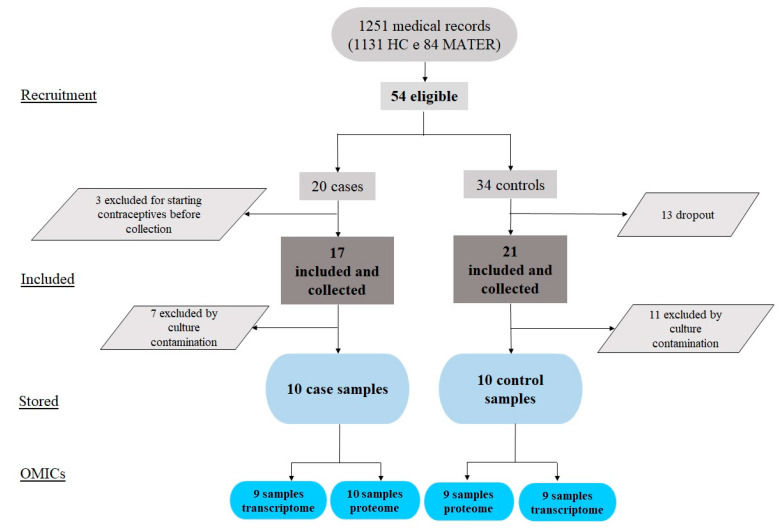
Study flowchart.

From November 2014 to December 2016, 1251 medical records of women assisted in the Assisted Reproduction Program of the University Hospital of the Faculty of Medicine of Ribeirão Preto and the Reference Center for Women’s Health of Ribeirão Preto (MATER) were evaluated. Among these women, 54 were eligible, 20 for the endometriosis group and 34 for the control group. After the interviews, 11 women in the control group withdrew from participating, and 2 we excluded due to irregular menstrual cycles. Furthermore, three were excluded from the endometriosis group because they had started hormonal treatment before collection. Thus, menstrual flow samples were effectively collected from 38 women. After sample exclusion for cell culture contamination, we stored ten samples from each group, the healthy and endometriosis, in the biorepository until use. For the large-scale approaches, we were unable to evaluate three transcript samples for low RNA integrity and one protein sample due to insufficient concentrations (Figure 1).

The clinical characterization of the women involved in this study and the establishment of the MenSCs in vitro model were previously described in [33]. No significant differences were observed regarding the patients’ clinical data, such as age, body mass index, and days in the menstrual flow collection. There were also no differences between the percentages of immunophenotypically labeled cells between the two conditions [33] (p. 736) (Table 1 and Table 2) with the expressions in agreement with the minimum criteria that define multipotent MSCs [34] and the MenSCs profile previously described [3,35,36].

### 2.2. Differential Transcript Profile

**Figure 2:**
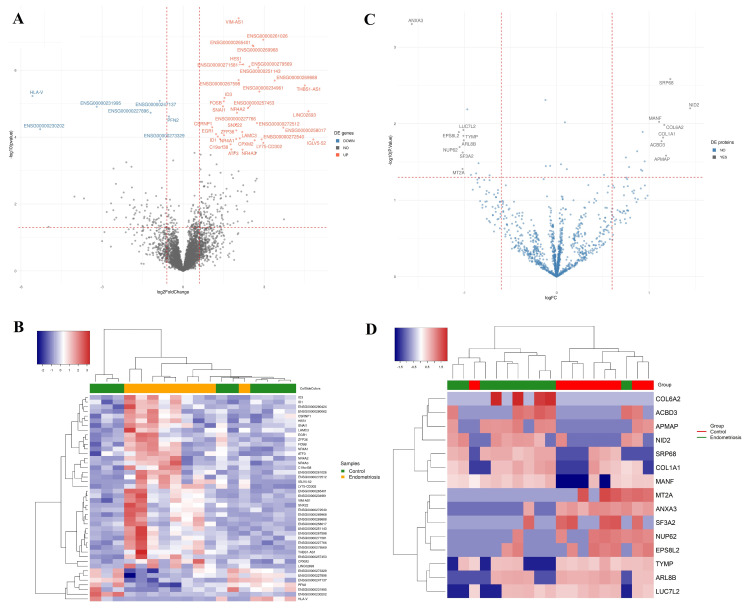
Differential expression data in graphical representation. Volcano plots showing gene (**A**) and protein (**C**) expressions in endometriosis MenSCs. Heatmap and hierarchical clustering of the DEGs (**B**) and DEPs (**D**) in MenSCs of women with endometriosis and healthy controls. Notes: Rows represent genes/proteins and columns represent samples.

RNA-seq analysis was performed to profile MenSC transcripts from women with and without endometriosis. The median mapping percentage was 77% (range from 60.6–88.6), with a median number of mapped reads of 45,818,461.5 (range 32,252,535 from to 73,828,997). We identified approximately 16,383 characterized transcripts in the database, 41 of which were differentially expressed genes (DEGs) (Supplementary Table S1, Figure 2A, FDR < 0.1, no FC cut-off). A greater homogeneity in the DEGs profile was observed among women with endometriosis than in the healthy group (Figure 2B). We also evaluated the predicted association networks between the 19 DE coding genes (see in https://version-11-5.string-db.org/cgi/network?networkId=b2VpX4zPkdlf) and verified whether they were previously associated in the literature with endometriosis (Table 1). The protein-protein interaction enrichment was strong (*p*-value < 1.0 × 10^−16^) between eleven proteins (represented here by *HES1*, *ATF3, ID1, ID3, FOSB, SNAI1, NR4A1, NR4A2, NR4A3, EGR1,* and *ZFP36* genes). It means that these proteins have more interactions with each other than expected for a random set of proteins and thus can be considered, at least partially, biologically connected as a group [37]. They are involved in pathways such as positive regulation of cell population proliferation, cell migration, response to a steroid hormone, regulation of epithelial cell proliferation, signaling by receptor tyrosine kinases, and others. Moreover, in the endometriosis scenario, the genes *ATF3, ID1, ID3, FOSB, SNAI1, NR4A1, EGR1,* and *ZFP36* were already associated with different disease aspects.

### 2.3. Differential Protein Profile

Our large-scale proteome approach identified 1373 proteins represented by two or more peptides. Among the proteins found, we analyzed the protein–protein interaction (PPI) of 34 proteins with a *p*-value < 0.05 (see in https://version-11-5.string-db.org/cgi/network?networkId=bNWNnkCu2eRF). The interaction enrichment was strong (*p*-value < 1.27 × 10^−7^) between seven proteins (SERPINH1, LEPRE1, FKB10, COL1A1, COL6A2, LAMA5, and NID2). They represent pathways related to the extracellular matrix organization, collagen formation, matrix metalloproteinases, and negative regulation of post-translational protein modification and serpin h1. Although the remaining proteins do not have a strong protein–protein interaction, interestingly, 17 of them play a role in acetylation processes. However, we considered differentially expressed proteins (DEPs) in endometriosis, those with a *p*-value < 0.05, and a 2-fold chance cut-off (Figure 2C,D). Among the 15 DEPs, COL1A1, COL6A2, and NID2 are among the proteins with strong interactions, and MT2A, TYMP, COL1A1, and COL6A2 have already been associated with endometriosis (Table 2).

### 2.4. The Biology of Proteomic and Transcriptomic Systems in Endometriosis MenSCs Reveal Related Pathways

**Figure 3:**
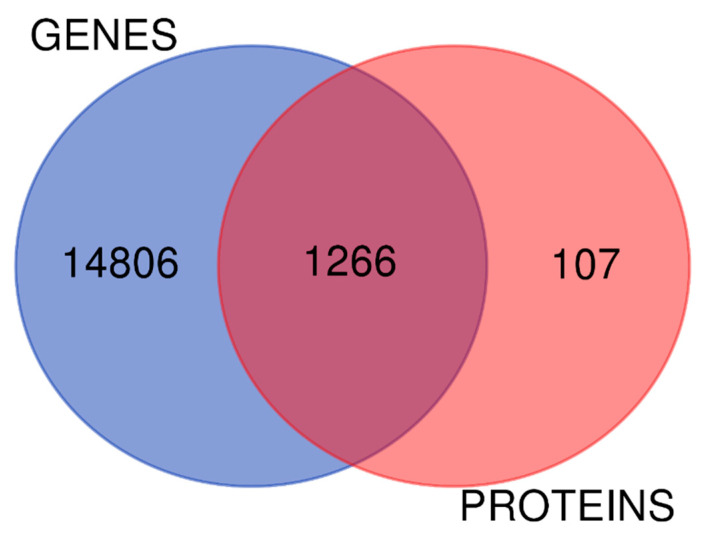
Venn diagram representing the set of identified proteins and the coding genes.

**Figure 4:**
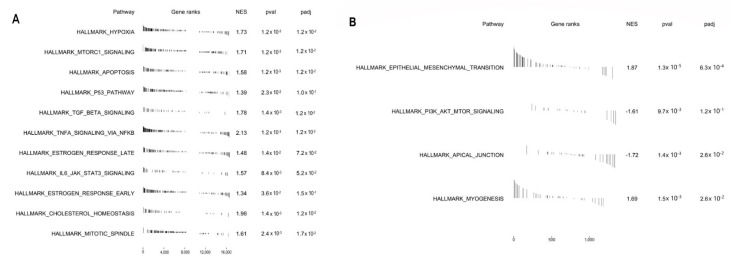
Enriched hallmark pathways identified by pre-ranked gene/protein set of the transcriptome (**A**) and the proteome data (**B**). The horizontal axis represents the gene set rank according to their enrichment scores. NES (normalized enrichment scores) with positive or negative signs indicate the direction of the enrichment pathway, that is, up-regulated or down-regulated genes.

We drew a Venn diagram to detect how many identified proteins were also present in the transcriptome (Figure 3). Overall, 92% of the identified proteins are represented at the transcriptional level. Interestingly, the network interaction obtained from the 8% of proteins detected exclusively in the proteomics data enriched the pathways involved in ncRNA metabolic processing, gene expression regulation, and epigenetics (see in https://version-11-5.string-db.org/cgi/network?networkId=bU6ZCYtP8bFU). Moreover, the agreement between these two biological systems is 52% regarding positive or negative signs of the logFC in the disease condition (Supplementary Table S2). Our enrichment analysis reveals signaling pathways that are modulated in an integrated form, such as PI3K signaling via AKT to mTORC1, mTORC1 signaling, epithelial–mesenchymal transition, hypoxia via HIF1A targets, TNFA signaling via NFkB, IL6 STAT3 signaling during acute phase response, and TGF beta signaling (Figure 4, Supplementary Table S3).

## 3. Discussion

To our knowledge, no study has applied the multi-omics approaches in menstrual blood-derived mesenchymal stem cells in the endometriosis condition. Here, we describe the transcriptome and proteome profile of these progenitor cells, highlighting dysregulated signaling pathways that modulate biological processes involved in angiogenesis, proliferation, cell migration, and inflammatory response. Furthermore, among the DEGs and DEPs, we emphasize *ATF3, ID1, ID3, FOSB, SNAI1, NR4A1, EGR1*, *LAMC3,* and *ZFP36* genes and MT2A, TYMP, COL1A1, COL6A2, and NID2 proteins that, when dysregulated in MenSCs, may play a role in the etiopathogenesis of endometriosis. All these molecules have already been linked in the literature with the disease (Table 1 and Table 2).

Although the relationship between the cellular components of menstrual flow and the etiopathogenesis of endometriosis is undeniable [55], as well as the immunoregulatory roles of MenSCs as actors of endometriosis [8], the origin of ectopic tissue remains unclear. In this scenario, more outstanding efforts have been devoted to unraveling molecular changes in progenitor cells in the endometrium (eMSC) [27,28,29,30] than those in menstrual blood (MenSCs) [7,31]. In summary, these papers show that resident endometrial mesenchymal stem cells (eMSCs) are precursors of endometrial stromal fibroblasts (eSFs) and that, although there is a progressive reduction in the number of differential expression genes time-dependent on cell culture exposure, the most remarkable differences are between cell populations (eMSCs versus SFs) rather than between endometriosis versus controls. Despite the heterogeneity in the study designs making interpretations difficult, our results are consistent with the scientific community: MenSCs present subtle, primary alterations in endometriosis.

Here, we profiled the transcriptome and proteome of MenSCs from women with endometriosis (*n* = 10) and without endometriosis (*n* = 10) and presented 19 DE coding genes and 15 DE proteins. We highlighted the genes *ATF3, ID1, ID3, FOSB, SNAI1, NR4A1, EGR1, LAMC3,* and *ZFP36* and proteins COL1A1, COL6A2, and NID2 as overexpressed and MT2A and TYMP proteins downregulated. The COL1A1 protein is the main collagen present in ectopic lesions and is suggested to participate in the progression of fibrotic diseases and wound healing [52,56], while TYMP, MT2A, and COL6A2 proteins were related to angiogenic potential and cellular proliferation [50,51,52,53]. Similar to proteins, apparently, the genes appear to enrich pathways related to apoptosis, angiogenesis, response to steroid hormones, migration, differentiation, and proliferation [33]. Furthermore, *ATF3* and *NR4A1* play a role in the process of endometrial decidualization and in the epithelial–mesenchymal transition [45,57], while *EGR1, ZFP36, ID1*, and *ID3* play an important role in the processes of inflammation associated with endometriosis as well as the ability to interact with TNFA and hypoxia [58,59].

Still considering the large-scale approaches, multi-omics technologies have substantially revolutionized endometriosis research [32]. They stratify the various biological scenarios and increase the resolution of the molecular analyses involved in the disease [19]. An interesting observation of ours obtained from the integration of gene–protein data was that 8% of the proteins (not represented in the transcripts) -enriched processes related to regulating gene expressions, such as ncRNA metabolism and epigenetic mechanisms. It may suggest a possible post-transcriptional regulatory effect on endometriosis MenSCs, mechanisms already well-described in the disease development [60,61]. In a recent study of our group, we found an upregulation of miR-200b-3p in endometriosis MenSCs from the same set of women studied here. We discussed that this change might lead to increased cell proliferation, stemness, and accentuated mesenchymal–epithelial transition process [33].

Additionally, our comprehensive functional enrichment analysis reveals integrated modulating signaling pathways, such as epithelial–mesenchymal transition (EMT) (↑) and PI3K signaling via AKT to mTORC1 (↓ in proteome), mTORC1 signaling, TGF beta signaling, TNFA signaling via NFkB, IL6 STAT3 signaling, and response to hypoxia via HIF1A targets (↑ in transcriptome). This related modulation of pathways makes sense when we think about the angiogenic, proliferative, migratory, and immunomodulatory potential of MenSCs in inflammatory conditions [1,62].

The PI3K/Akt/mTOR pathway has already been reported by other researchers in endometriosis [63,64,65] and in cancer due to its involvement with mechanisms responsible for tumor progression [66,67]. Endometriosis is known to be a benign disease, but it shares characteristics with cancer, such as resistance to apoptosis, invasion, angiogenesis, and generating a chronic inflammatory environment [68]. In our results, the PI3K/AKT/mTOR pathway was enriched with down-regulated proteins, and this may reflect the active mTORC1 pathway in the transcriptome since its hyperactivation can lead to feedback inhibition of PI3K/AKT signaling [69]. Kim et al. [70] reported that mTORC1 has elevated activity in most tumors due to its potential for activating oncogenes and inactivating tumor suppressors and that TGF-B may mediate glucose response through PI3K/AKT/mTOR signaling through induction by inflammatory cytokines through the expression of the HIF1A protein [71].

Consistent with other studies, the TGF-B pathway is increased in women with endometriosis and the response to hypoxia via HIF1A targets. Studies have identified an abundance of TGF-B in the peritoneal fluid of women with the disease, which may come from shed endometrial tissue, ectopic endometrial cells, and circulating and increased macrophages in endometriosis [72,73]. Furthermore, hypoxic conditions are related to angiogenesis during the development of endometriotic lesions as well as elevated levels of pro-inflammatory cytokines such as TNFA and the *ID1* and *ID3* genes [74]. Moreover, the dysregulation of the IL6/STAT3 signaling pathway in endometriosis can also be induced under hypoxia conditions and in the presence of inflammatory cytokines such as TNFA, contributing to the establishment of ectopic tissue [75]. Another study with peritoneal fluid from women with endometriosis showed that TNFA signaling could increase *EGR1* expression and collaborate with establishing and maintaining the disease [76]. In addition, TNFA can be increased by NFkB, accelerating the prolonged inflammatory process responsible for the reduction of apoptosis activity in the disease [77], making sense for TNFA signaling via the NFkB pathway in our findings.

The EMT pathway has been widely associated with endometriosis [48,78,79]. It is noteworthy that it can be induced by inflammatory cytokines such as TGF-B and TNFA and under hypoxic conditions [80]. Other studies have shown that PI3K/Akt/mTOR is also capable of inducing EMT as well as the important involvement of *SNAIL* in this process [48,81,82]. We believe that the MenSCs (protein phenotype) are genetically programmed (transcriptome) for accentuated mesenchymal–epithelial transition in endometriosis [33].

We observed that the pathways enriched in our study are similar to the literature. This fact only reinforces the idea that proteomic analysis (phenotype) complements the data observed in the transcriptome (genetic programming), but it also leads to an improved understanding of the MenSCs mechanisms acting in endometriosis.

These pathways are related to inflammatory processes. In a meta-analysis, our group discusses the same pathways in the endometrium of women with endometriosis and associates their imbalances directly with macrophage polarization (M1 to M2) and disease progression [26]. This polarization has been found in tumor conditions and is believed to be stimulated by the tissue microenvironment resulting from hypoxia, NFkB signaling alterations, and in the composition of the extracellular matrix (ECM). Thus, when polarized, it promotes immune system escape, angiogenesis, and metastasis [83].

The endometrium contains a variety of immune cells involved in tissue repair that are subject to changes during the menstrual cycle. It is known that there is a continuous increase in the number of macrophages, peaking at the desquamation phase, which suggests an inflammatory component to menstruation [84,85]. An in vitro study reported that mesenchymal cells derived from endometrioma induce macrophages to modulate evasion of the immune system allowing lesion growth [86]. Therefore, we believe that the inflammatory microenvironment of women with endometriosis can promote macrophage polarization, affecting the molecular signature of MenSCs and thus contributing to the origin and maintenance of the disease.

The most significant strength of our study is that we used integrative omics approaches in the same biological condition. Thus, even under subtly dysregulated expression conditions, it was possible to interpret genetic programming with the temporally regulated protein phenotype. Further, we used stringent inclusion criteria to define the biological groups as homogeneously as possible. Despite the advantages, this study had some limitations. The expression profile after the culture models must be carefully interpreted. It is not easy to extrapolate the same results to in vivo systems, as the culture systems can mask the cellular environment [28]. It is also important to remember that these results must be validated in larger sample sizes, including endometriosis I–II and III–IV.

## 4. Materials and Methods

### 4.1. Ethics Statement and Duration

This case-control study was conducted under the approval of The Research Ethics Committee of the University Hospital of the Ribeirao Preto Medical School (HCRP 3644/2019) from April 2019 to December 2021. All participants provided written informed consent. The cells used in this study were collected from November 2014 to December 2016 (HCRP 15227/2012) following the ethics guidelines established by the Declaration of Helsinki and were transferred to a biorepository (HCRP 3644/2019) in the Human Reproduction Sector of the Department of Gynecology and Obstetrics of the Ribeirão Preto Medical School.

### 4.2. Settings

We included the recruited women from the Assisted Reproduction Program of the University Hospital of the Ribeirao Preto Medical School and the Reference Center for Women’s Health in Ribeirao Preto (MATER). The number of patients per group is presented in Figure 1. Samples were processed, and the in vitro model was established at the Hemotherapy Center of Ribeirão Preto. The RNA-seq protocols were performed at the Center for Genomic Medicine of the Ribeirao Preto Medical School and the Proteomic at the Translational Medicine Drug Research and Development Center of the Federal University of Ceará. We conducted bioinformatics analysis at the Laboratory for Translational Data Science of the Department of Gynecology and Obstetrics and the Department of Biochemistry and Immunology of Ribeirão Preto Medical School.

### 4.3. Participants and Eligibility Criteria

The clinical characterization of the women involved in this study was previously described in [33] (p. 736, Table 1). In summary, eligible patients were women between 18 and 40 years of age with regular menstrual cycles (intervals from 24 to 32 days ± three days; 2 to 7 days of duration) and not on hormone therapy for at least three months before the sample collection. Any uterine disorder, systemic disease, tumor, endocrinopathy, or cardiovascular or rheumatological diseases were excluded. The case group was composed of women with a histological and laparoscopic diagnosis of endometriosis classified as III or IV [87]. We selected patients who had undergone surgical treatment an average of 6 years (SD ± 3.7) before collection. Since the stem cells have tropism for endometriotic lesions [88], we selected patients who had presented diagnostic imaging suggestive of endometrioma at the time of collection as evidence of active disease in the pelvic cavity. For the control group, we included fertile women (with children and no history of recurrent abortion) without clinical symptoms of endometriosis and endometriotic lesions by laparoscopy.

### 4.4. Characterization and Establishment of the MenSCs In Vitro Model

**MenSCs. Sample collection**. The menstrual blood was collected through a silicone cup (Inciclo, São Paulo, Brazil) sterilized with gamma radiation. It was inserted into the vagina for 3 h during the second, third, or fourth day of the menstrual cycle. The samples were stored at 4 °C for up to 4 h in a solution containing PBS 1× (ThermoFisher, Waltham, MA, USA), antibiotic-antimycotic solution 10× (Gibco, Waltham, MA, USA), and 10% acid citrate-dextrose (JP Farma, São Paulo, Brazil). ***MenSCs isolation***. We adopted the isolation method described by [4] with modifications [89]. The mononuclear cell layer was isolated by density gradient centrifugation at 800× *g* for 30 min at 22 °C with Ficoll-Paque (#71-7167-00AG, GE Healthcare Bio-Sciences, Sweden). The cells were cultivated to α-minimum essential medium (# 11900-016, Gibco, Waltham, MA, USA) with 15% fetal bovine serum (# SH30071.03, GE Healthcare-HyClone, Waltham, MA, USA), 1% penicillin/streptomycin (# 15140-122, Gibco, Waltham, MA, USA), 10 mM HEPES (# H4034, Merck, Darmstadt, Germany), and 20 mM sodium bicarbonate (# 56297, Merck, Darmstadt, Germany). We sub-cultured the cells using 0.05% trypsin-EDTA solution (#25300054, Gibco, Waltham, MA, USA). **Cells characterization.** Following the minimal criteria for multipotent mesenchymal stem cells [34], the MenSCs were characterized for expressing 23 markers following the manufacturer’s instructions on the FACSCalibur flow cytometer (BD Biosciences, Waltham, MA, USA) using CellQuest™ version 4.0 software (BD Biosciences, Waltham, MA, USA) and for their ability to differentiate into adipocytes and osteocytes. We previously published these protocols and results in [33] (p. 736, Table 2, and Supplementary Figures S1 and S2). We sub-cultured the cells until passage 3 (P3) for cell characterization analysis (early culture). However, the cells were stored in P2 in the biorepository (HCRP 3644/2019). We expanded the cells from P2 to P3 for transcriptome and proteome analyzes.

### 4.5. OMICs Approaches

#### 4.5.1. Transcriptome: Total RNA Extraction, RNA Integrity, and Quantification

Total RNA was extracted using the AllPrep DNA/RNA/miRNA Universal Kit (#80224, Qiagen, Waltham, MA, USA) according to the manufacturer’s instructions, followed by treatment with Ambion DNA-free Kit (#AM1906, Invitrogen, Waltham, MA, USA) for removal of contaminating DNA. We assessed the RNA integrity using Agilent RNA 6000 Nano Kit (# 5067-1511, Agilent, Waltham, MA, USA) in the 2100 Bioanalyzer Instrument (Agilent, Santa Clara, CA, USA), and we included only samples with RNA Integrity Number (RIN) ≥ 8 for library preparation. Total RNA concentration was measured using Qubit RNA BR Assay Kit (#Q10210, Invitrogen, Waltham, MA, USA) on the Qubit 2.0 Fluorometer (ThermoFisher, Waltham, MA, USA). **Library construction**. Ribosomal RNA-depleted strand-specific RNA libraries were generated with TruSeq Stranded Total RNA LT Sample Prep Kit (with Ribo-Zero Gold) set A (#RS-122-2301) and set B (#RS-122-2301) (Illumina, San Diego, CA, USA) following the manufacturer’s instructions. **Clustering and running**. The library was sequenced on NextSeq 500 System (Illumina, San Diego, CA, USA) using chemistry v2 with NextSeq 500/550 High Output Kit (#FC-404-2004, Illumina, San Diego, CA, USA) in paired-end mode with a read length of 2 × 150 bp. The paired-end sequencing runs were performed containing six samples each (3 controls and 3 cases per run), distributed in four lanes. We realized three sequencing runs. Upon run completion, libraries were demultiplexed, adapters trimmed, and fastq files were generated using the Illumina NextSeq Control Software version 2.02 on BaseSpace (Illumina’s cloud-based resource). **Raw data processing and statistics.** The reads in fastq files were trimmed based on the quality score <20, and the quality control was evaluated using the fastqc software (Illumina NextSeq Control Software version 2.0.2 on BaseSpace). Gene-level read counts were inferred using the pseudo-alignment method using the Salmon software with default parameters and the GRCh38 reference genome based on the Ensemble annotation [90]. Differential expression analysis was performed using the DESeq2 pipeline [91] based on the coefficient of variation between the two conditions (control versus endometriosis) and adjusted by the mean percentage (z-score adjusted) of cells expressing the CD73 and CD90 proteins at the flow cytometry analysis. Genes with very low expression (sum of reads for all samples <5) were filtered out of the statistical analysis. We considered differentially expressed genes (DEGs) if adjusted *p*-value by the false-discovery ratio (FDR) <0.1 (without FC cut-off). After normalization with the rlog() function using the blind parameter (the dispersion estimation is unbiased by the information about experimental groups) in the DESeq2 package in R, the differentially expressed genes were selected and used for agglomerative hierarchical clustering. We used the Euclidean distance as the metric for dissimilarity and the complete agglomerative method for clustering. The dendrograms were plotted with the heatmap scaling (Z-score) the expression values by rows using the heatmap3 package in R [92] (https://CRAN.R-project.org/package=heatmap3, accessed on 1 June 2021).

#### 4.5.2. Shotgun Proteomic: Extraction, Quantification, Trypsinization, and Desalination of Proteins

Total proteins were extracted and solubilized using cell lysis buffer containing 8M urea, 10 mM Tris (pH = 8.0), and protease inhibitor (#P8340, Sigma-Aldrich, Darmstadt, Germany). Then, 3 sonication cycles at 45 W for 5 min each were performed. The samples were centrifuged at 20,000× *g* for 30 min at 4 °C, and total proteins were quantified in triplicates by the Bradford method [93] using the Quick Start Bradford Protein Assay Kit (#15000201, Bio-Rad, Hercules, CA, USA). Approximately 100 μg of total proteins were reduced by incubation at 37 °C for 30 min with 10 μg/μL dithiothreitol (DTT) (#D9779, Sigma-Aldrich, Darmstadt, Germany). Subsequently, they were alkylated at 25 °C and in light deprivation for 30 min by the addition of 10 μg/μL iodoacetamide (IAA) (#I1149, Sigma-Aldrich, Darmstadt, Germany). Then, the samples were diluted 8x with 10 mM Tris solution (pH = 8.0). **Protein digestion**. Total proteins were incubated with trypsin (#V5111, Promega, Madison, WI, USA) and diluted in 20 mM Tris (pH = 8.0) in a 1:50 ratio (enzyme/proteins, m/m) for 18 h at 37 °C under agitation. The peptides obtained from the digested samples were purified on reversed-phase OASIS-HLB columns (#186000383, Waters, Framingham, MA, USA). After the OASIS-HLB columns, the peptides were dried in the SpeedVac apparatus (Thermo Scientific, Marietta, OH, USA), resuspended in the appropriate buffer, and quantified by the Qubit Protein Assay Kit (#Q33211, ThermoFisher, Waltham, MA, USA). **UHPLC-MS/MS detection.** The peptides were fractionated in UHPLC Dionex Ultimate 3000 (ThermoFisher, Waltham, MA, USA) and analyzed in the Q Exactive Plus HMR mass spectrometer (ThermoFisher, Waltham, MA, USA) in full MS/ddMS2 (Top5)-positive mode. One µg of peptides per sample was injected into the mass spectrometer for 210 min using 18 0.1% formic acid (solvent A or equilibrium) and 0.1% acetonitrile/formic acid in an 80:20 ratio (solvent B or elution). The elution followed an optimized linear solvent B gradient from 4 to 85%. **Raw data processing and statistics.** Raw files from MS analysis were processed using the MaxQuant computational proteomics platform [94] version 1.6.17.0, which obtained a list of identified proteins and the relative label-free quantification (LFQ). The search parameters used were carbamidomethylation of cysteine residues as a fixed modification; oxidation of methionine residues as a variable modification, trypsin enzyme with a tolerance of two miss cleavages; mass error tolerance for precursor peptide of 20 ppm in the first search and 6 ppm in the main search; and mass tolerance for fragments (MS/MS) of 0.5 Da, false-discovery rate (FDR) of 1% for proteins and peptides. The LFQ was normalized manually based on total ion intensity for each LC-MS/MS run and considered at least 1 peptide identified by MS/MS for paired comparisons. The statistical analyses were performed with normalized intensity values (LFQ intensity) using the Limma package [95] in the R environment, considering only proteins identified with 2 or more peptides and reviewed by the UniProt database (https://www.uniprot.org/, accessed on 20 February 2022). For constructing the contrast matrix, we considered the outcomes of interest (endometriosis and healthy control). It was investigated whether weighting the proportion of mesenchymal cells typically labeled with CD90 and CD73 influenced the results, but this was not observed. Finally, we used a linear model through the lmFit function and considered the fit patterns. We also evaluated the interference of two fitting methods, namely “least square” and “robust regression”, without significant differences, so we kept the default fit. After the linear fit, we used the eBayes function that applies an empirical Bayesian method to moderate the t-statistic. We set the cut-offs at <5% for *p*-value and at 2.0 for fold-change (FC) to consider differential expression proteins (DEPs).

### 4.6. Enrichment Analysis

The genes and proteins obtained from the large-scale approaches were evaluated using public databases and free, open-source software. First, we used the STRING database v11.5 to summarize the predicted association networks for proteins (with a *p*-value of <0.05) and genes (with an adjusted *p*-value of <0.1) set at a medium confidence score (0.400) [37]. We also drew a Venn diagram using a web tool (http://bioinformatics.psb.ugent.be/webtools/Venn/, accessed on 12 March 2022) to detect how many identified proteins were represented in the transcriptome. We used all coding genes for this diagram.

As statistically significant gene expressions and arbitrarily determined cut-offs do not always represent biological variations [26], we performed the pathway enrichment analysis of all genes and proteins pre-classified by logFC without filtering. We used the *Molecular Signatures Database* (MSigDB) v7.5.1, hallmark gene sets collection, available in the Gene Set Enrichment Analysis (GSEA) web tool [96]. We dictated that the parameters included 1000 permutations and weighted enrichment statistics (*p*-value = 0.05). The *p*-value of <5% and FDR of <10% were considered significant. We considered the positive or negative signs of the normalized enrichment scores (NES) to interpret the direction of the pathway. More details can be obtained by consulting documentation at http://www.gsea-msigdb.org/gsea/index.jsp, accessed on 12 May 2022.

### 4.7. Statistics

Exploratory data analysis was performed using descriptive statistics. Clinical variables and immunophenotypic markers were compared between groups using the Mann–Whitney test (independent samples). Analyses were performed in SAS software, version 9.4. For interpretation, a *p*-value of <5% was considered significant.

## 5. Conclusions

Our findings highlight primary changes in the endometriosis MenSCs that may favor the tissue implantation at the ectopic site. The global expression profile enriched critical pathways already related to the endometriosis condition, such as PI3K signaling via AKT to mTORC1 [63,64,65], mTORC1 signaling [97], TNFA signaling via NFkB [76,77], IL6 STAT3 signaling [75], TGF beta signaling [72,73], and hypoxia via HIF1A targets [74]. These dysregulations suggest that the chronic inflammatory endometrial microenvironment [26] can modulate these cells and provide opportunities for the etiopathogenesis of the disease. Our results are important for identifying challenges and opportunities for future research and leveraging knowledge in regenerative and precision medicine in this disease.

## Figures and Tables

**Table 1:** Differential expression of coding genes in endometriosis MenSC and endometriosis-related genes/proteins in the literature.

Ensemble ID.	Official Gene Symbol	Gene Type	Chromosome Location	Gene Name	Log2fc	*p*-Value	P-Adj	Endometriosis-Related Gene/Protein in the Literature
ENSG00000162772	*ATF3*	Protein coding	1q32.3	Activating transcription factor 3	1.782043	0.000234	0.099158	[38]
ENSG00000214212	*C19orf38*	Protein coding	19p13.2	Chromosome 19 open reading frame 38	1.756059	0.000165	0.073847	
ENSG00000121898	*CPXM2*	Protein coding	10q26.13	Carboxypeptidase X, M14 family member 2	2.647503	0.000124	0.058576	
ENSG00000144655	*CSRNP1*	Protein coding	3p22.2	Cysteine and serine-rich nuclear protein 1	1.076952	5.07 × 10^−5^	0.036267	
ENSG00000120738	*EGR1*	Protein coding	5q31.2	Early growth response 1	1.217478	8.09 × 10^−5^	0.049207	[39]
ENSG00000125740	*FOSB*	Protein coding	19q13.32	FosB proto-oncogene, AP-1 transcription factor subunit	1.50465	8.48 × 10^−6^	0.009987	[26,40]
ENSG00000114315	*HES1*	Protein coding	3q29	Hes family bHLH transcription factor 1	2.103473	5.75 × 10^−7^	0.001951	
ENSG00000125968	*ID1*	Protein coding	20q11.21	Inhibitor of DNA binding 1, HLH protein	1.282674	9.56 × 10^−5^	0.054622	[41,42]
ENSG00000117318	*ID3*	Protein coding	1p36.12	Inhibitor of DNA binding 3, HLH protein	1.530359	6.71 × 10^−6^	0.009036	[41,42]
ENSG00000211643	*IGLV5–52*	Protein coding	22q11.22	Immunoglobulin Lambda Variable 5–52	4.818943	0.000117	0.056648	
ENSG00000050555	*LAMC3*	Protein coding	9q34.12	Laminin subunit gamma 3	2.187422	6.99 × 10^−5^	0.043905	[43,44]
ENSG00000248672	*LY75-CD302*	Protein coding	2q24.2	LY75-CD302 readthrough	2.969177	0.000148	0.068122	
ENSG00000123358	*NR4A1*	Protein coding	12q13.13	Nuclear receptor subfamily 4 group A member 1	1.965574	0.000114	0.056648	[45]
ENSG00000153234	*NR4A2*	Protein coding	2q24.1	Nuclear receptor subfamily 4 group A member 2	1.993967	1.86 × 10^−5^	0.015948	
ENSG00000119508	*NR4A3*	Protein coding	9q31.1	Nuclear receptor subfamily 4 group A member 3	2.200431	0.000237	0.099158	
ENSG00000124216	*SNAI1*	Protein coding	20q13.13	Snail family transcriptional repressor 1	1.473873	1.20 × 10^−5^	0.012836	[46,47,48,49]
ENSG00000157734	*SNX22*	Protein coding	15q22.31	Sorting nexin 22	1.968101	6.28 × 10^−5^	0.040804	
ENSG00000128016	*ZFP36*	Protein coding	19q13.2	ZFP36 ring finger protein	1.512248	8.75 × 10^−5^	0.05156	[39]
ENSG00000070087	*PFN2*	Protein coding	3q25.1	Profilin 2	−0.52405	2.42 × 10^−5^	0.019852	

**Table 2:** Differential expression proteins (DEPs) in endometriosis MenSCs and endometriosis-related genes/proteins in the literature.

Official Protein Symbol.	Chromosome Location	Protein Name	Log2fc	*p*-Value	Endometriosis-Related Gene/Protein in the Literature
ANXA3	4q21.21	Annexin A3	−1.57	0.00050	
EPS8L2	11p15.5	EPS8 like 2	−1.06	0.01287	
NUP62	19q13.33	Nucleoporin 62	−1.05	0.02018	
MT2A	16q13	Metallothionein 2A	−1.02	0.03902	[50]
SF3A2	19p13.3	Splicing factor 3a subunit 2	−1.02	0.02382	
TYMP	22q13.33	Thymidine phosphorylase	−1.01	0.01438	[51]
LUC7L2	7q34	LUC7 like 2, pre-mRNA splicing factor	−1.01	0.01209	
ARL8B	3p26.1	ADP ribosylation factor-like GTPase 8B	−1.01	0.01648	
MANF	3p21.2	Mesencephalic astrocyte-derived neurotrophic factor	1.11	0.00944	
ACBD3	1q42.12	Acyl-CoA binding domain-containing 3	1.14	0.01693	
COL1A1	17q21.33	Collagen type I alpha 1 chain	1.15	0.01517	[52]
COL6A2	21q22.3	Collagen type VI alpha 2 chain	1.16	0.01035	[53]
APMAP	20p11.21	Adipocyte plasma membrane-associated protein	1.18	0.02613	
SRP68	17q25.1	Signal recognition particle 68	1.23	0.00261	
NID2	14q22.1	Nidogen 2	1.45	0.00629	[54]

## Data Availability

The RNA-sequencing data is available in the repository Sequence Read Archive (SRA) of the National Center for Biotechnology Information (NCBI) (Permanent link: http://www.ncbi.nlm.nih.gov/bioproject/884641), study number: PRJNA884641. The mass spectrometry proteomics data have been deposited to the ProteomeXchange Consortium via the PRIDE partner repository with the dataset identifier PXD037033.

## Supplementary Materials

The supporting information can be downloaded at: https://www.mdpi.com/article/10.3390/ijms231911515/s1.
